# Functionally Enhanced XNA Aptamers Discovered by Parallelized Library Screening

**DOI:** 10.1021/jacs.3c09497

**Published:** 2023-11-14

**Authors:** Adriana Lozoya-Colinas, Yutong Yu, John C. Chaput

**Affiliations:** † Department of Pharmaceutical Sciences, University of California, Irvine, Irvine, California 92697-3958, United States; ‡ Department of Chemistry, University of California, Irvine, Irvine, California 92697-3958, United States; § Department of Molecular Biology and Biochemistry, University of California, Irvine, Irvine, California 92697-3958, United States; ∥ Department of Chemical and Biomolecular Engineering, University of California, Irvine, Irvine, California 92697-3958, United States

## Abstract

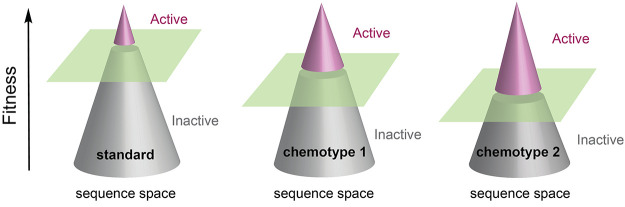

In vitro evolution strategies have been used for >30 years to generate nucleic acid aptamers against therapeutic targets of interest, including disease-associated proteins. However, this process requires many iterative cycles of selection and amplification, which severely restricts the number of target and library design combinations that can be explored in parallel. Here, we describe a single-round screening approach to aptamer discovery that relies on function-enhancing chemotypes to increase the distribution of high-affinity sequences in a random-sequence library. We demonstrate the success of de novo discovery by affinity selection of threomers against the receptor binding domain of the S1 protein from SARS-CoV-2. Detailed biochemical characterization of the enriched population identified threomers with binding affinity values that are comparable to aptamers produced by conventional SELEX. This work establishes a highly parallelizable path for querying diverse chemical repertoires and may offer a viable route for accelerating the discovery of therapeutic aptamers.

## Introduction

Darwinian evolution has long been viewed as the cornerstone of biology and the inspiration for laboratory methods capable of discovering and refining biopolymer function through iterative rounds of selection.^1−3^ Efforts to extend this paradigm to artificial genetic polymers, commonly known as xeno nucleic acids or XNAs, have afforded examples of biologically stable XNA frameworks that can fold into structures with specific ligand-binding and catalytic activities.^4−9^ One prominent example is α-l-threofuranosyl nucleic acid (TNA),^10^ a 2′,3′-linked genetic polymer that is invisible to nucleases that degrade DNA and RNA^11^ and is acid stable.^12^ However, despite favorable physicochemical properties, XNA-based technologies face the same inherent limitations as their natural counterparts.^13^ Functional molecules are discovered by in vitro selection, which is a time-consuming process that relies on iterative cycles of selection and amplification to identify high-activity sequences. Although machine learning algorithms could 1 day solve this problem,^14^ reinforcement learning requires large amounts of data that are difficult to generate using the traditional model of one target, one library, and many rounds of selection.

**Figure 1:**
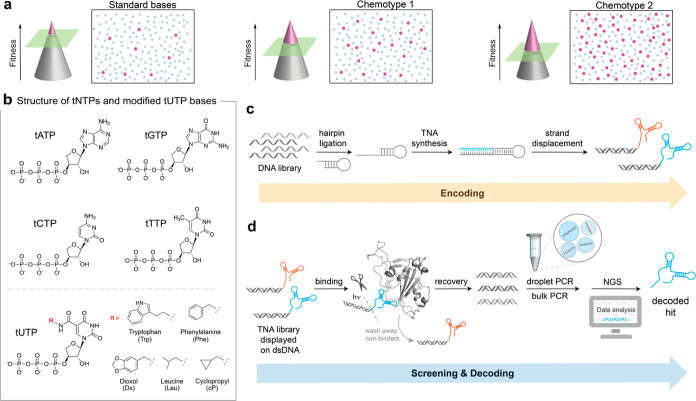
Aptamer screening. (a) Cartoon representation of the distribution of functional aptamers within a library. Libraries comprising function-enhancing chemotypes increase the abundance of active sequences relative to that of conventional standard base libraries. Color: binders (pink) and nonbinders (gray). Green plane denotes the threshold for the detection of binding affinity by an analytical technique. (b) Chemical structures of TNA triphosphates (tNTPs) used to generate (3′,2′)-α-l-TNA libraries using a laboratory-evolved TNA polymerase to transcribe DNA into TNA. Libraries are prepared with diverse chemotypes ranging from standard bases only to uracil residues equipped with functionally diverse side chains. (c) Library preparation. DNA display establishes a genotype–phenotype relationship by covalently linking each TNA molecule to its encoded dsDNA template. (d) Library screening. The TNA library is incubated with the target protein and bound molecules are separated from the unbound pool by affinity capture on Ni-NTA beads. Information carrying DNA molecules is recovered by photocleavage, uniformly amplified, and subjected to high-throughput sequencing (HTS).

The requirement for recursive rounds of selection reflects the difficulty in surveying large pools of random sequences for members that can fold into structures that function with a predefined activity. A nucleic acid library consisting of 40 randomized positions, for instance, has a theoretical diversity of ∼10^24^ unique sequences, which is considerably larger than the number of sequences sampled in a typical aptamer selection (∼10^14^).^15^ Estimates on the frequency of folded and functional aptamers vary according to target but can be as low as 10^–10^, emphasizing the magnitude of the problem facing those attempting to bypass the evolutionary process.^16^ To investigate the feasibility of converting in vitro selection into a high-throughput screen, we considered the potential for function-enhancing side chains to increase the abundance of high-activity sequences in a random-sequence library (Figure 1a). We were inspired by the impact that base-modified libraries have made on the results of aptamer selections.^17−22^ In a large systematic study performed across a range of protein targets, it was discovered that aromatic side chains increased the success rate of aptamer selections from 30 to 84%, with 55% of the selections producing aptamers with a solution binding affinity constant (*K*_D_) of <1 nM.^23^ This finding is consistent with the ability of base-modified aptamers to effectively mimic the paratope surface of an antibody.^24^

Here, we wished to test the hypothesis that side chains could increase the abundance of functionally enhanced TNA aptamers, termed threomers, in a pool of random sequences (Figure 1a). To test this hypothesis, we envisaged the development of a realistic and highly parallelizable pathway for querying diverse chemical repertoires that could catalyze the expansion of XNA aptamers as tools for diagnostic and therapeutic applications. We began by designing a platform that would enable the comparison of conventional standard base (std) libraries to functionally enhanced libraries carrying amino-acid-like side chains at the C5 position of uracil bases in TNA molecules. In addition to the std library, separate TNA libraries augmented with leucine (Leu), cyclopropyl (cP), phenylalanine (Phe), dioxol (Dx), and tryptophan (Trp) chemotypes were generated using chemically synthesized TNA nucleoside triphosphates (Figure 1b).^25^ Our results indicate that a single round of high-throughput screening is sufficient to generate threomers that can bind their target protein with low to subnanomolar affinity and discriminate against off-target proteins known to bind nonspecifically to nucleic acid sequences.

## Results

**Figure 2:**
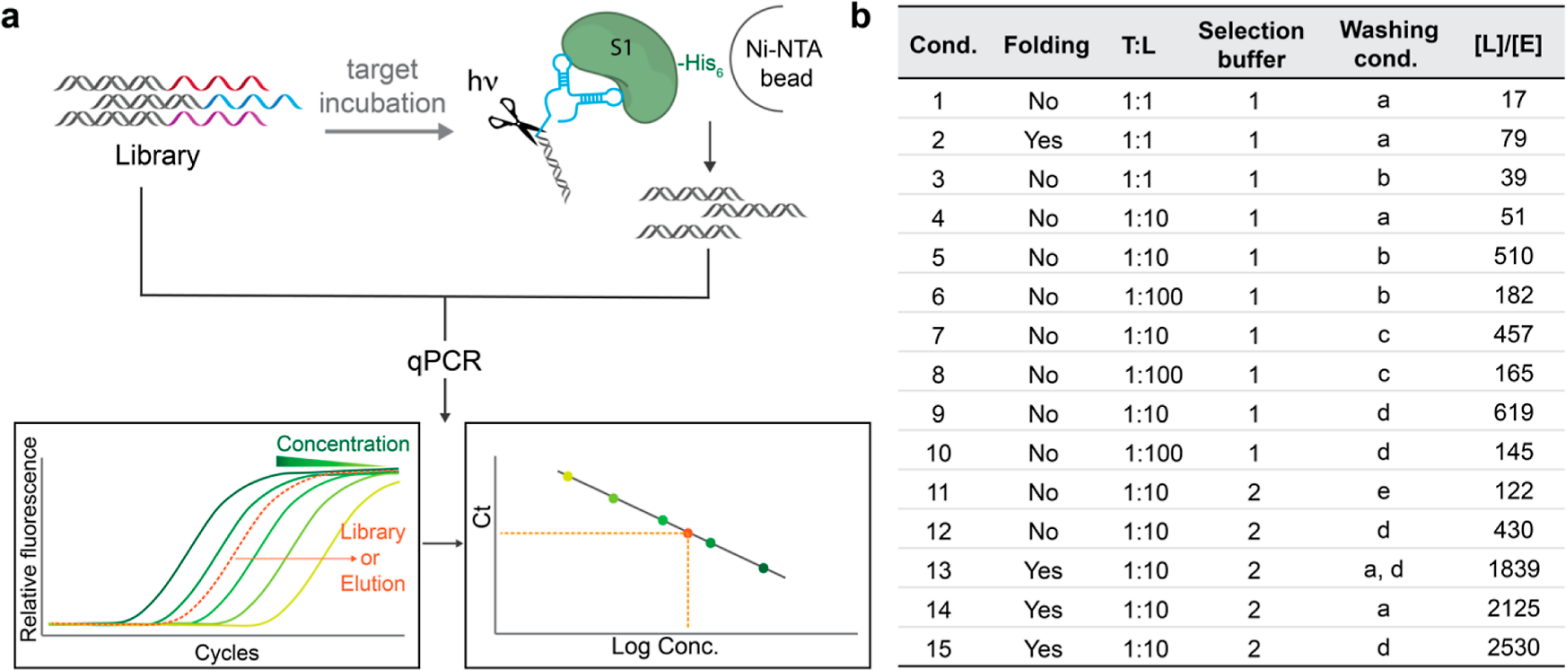
Selection conditions. (a) Parallelized single-round aptamer screening demands stringent binding conditions to partition functional and nonfunctional sequences. Cartoon representation of an evaluation of the binding and wash conditions for a starting library tested against S1. (b) Summary of the selection conditions evaluated to increase the separation of active and inactive sequences. Buffer 1: 25 mM Tris (pH 8.0) and 150 mM NaCl. Buffer 2: 10 mM HEPES (pH 7), 150 mM NaCl, 3 mM EDTA, 0.05% Tween 20, 0.5 mg/mL BSA, and 0.05 mg/mL ssDNA. Washing conditions refer to selection buffer supplemented with (a) 1 M NaCl, (b) 0.5 M urea, (c) 1 M urea, (d) 2 M urea, and (e) no additive. Library refolding involves heating to 65 °C and cooling on ice. *T*/*L* refers to target/library molar ratio and [*L*]/[*E*] denotes the molar ratio of the starting library and elution sequences.

Aptamer discovery based on a single round of protein target binding demands stringent binding conditions to partition functional sequences away from the nonfunctional pool. To probe the partitioning of active and inactive library members, we subjected a Phe chemotype library to a competitive binding assay that explored a broad range of conditions, which included aptamer refolding, target-to-library (*T*/*L*) ratios, and various buffer conditions for protein binding and bead washing. Accordingly, each library aliquot (10^12^ molecules) was passed through a negative selection step to remove sequences with affinity to Ni-NTA beads and a positive selection step for binding to a representative therapeutic protein (Figure 2a), which in this case was a His-tagged version of the SARS-CoV-2 spike glycoprotein subunit 1 (S1), chosen based on the known affinity of Phe-modified TNA aptamers to S1.^26^ Because the theoretical sequence space of this library (*N*_18_) is ∼6.9 × 10^10^, each library aliquot should contain a similar distribution of sequences. Molecules that bound to the S1 protein in solution were partitioned away from the unbound material by passing the mixture over Ni-NTA beads to capture the bound aptamer–protein complexes on a solid-support matrix. Following several wash steps, the dsDNA-encoding tags were photocleaved and quantified relative to their starting molar amount using a quantitative polymerase chain reaction (qPCR) (Figure S1). Selection conditions with higher library to elution values (*L*/*E*) were viewed as more optimal for a protein binding assay as the elution fractions were expected to be more enriched in functional aptamers than conditions with lower *L*/*E* values.

The results of our binding assays show the profound effect that the binding and wash conditions can have on the partitioning efficiency of functional aptamers from weakly active and inactive library members (Figure 2b). The inclusion of urea as a chaotropic agent in the wash buffer to destabilize noncovalent interactions between weakly folded library members and the S1 protein (conditions b, c, and d) increased the partitioning efficiency by 10-fold as compared to a high salt wash (condition a). Further, varying the target to library ratio (*T*/*L*) revealed that a 1:10 ratio was optimal for a naïve library. Surprisingly, the inclusion of bovine serum albumin (BSA) and salmon sperm DNA (ssDNA) as competitive proteins and nucleic acid agents, respectively, in the binding buffer (buffer 2) exhibited only a modest 2-fold increase in partitioning efficiency. However, the most striking difference (∼2500-fold partitioning) was observed with conditions 13–15, which combined the cumulative effects of these smaller changes to the standard binding and washing protocol with an aptamer refolding step. Control experiments show that the aptamer refolding step and urea wash did not disrupt the dsDNA region of the DNA display library (Figure S2). As such, condition 15, which included an aptamer refolding step, BSA, and ssDNA in the binding buffer, 10-fold less protein to the library, and urea in the wash buffer, was chosen as the optimal condition for our single-round aptamer screening approach.

Encouraged by the promising partitioning efficiency of the optimized binding and wash conditions, we chose to investigate the relationship between library chemotype and function by challenging each of the six chemotype libraries to bind a His-tagged version of the receptor binding domain of the S1 protein (S1-RBD) in a parallel binding assay. The screens were performed using a DNA-display library format (Figure S3) that simplifies the selection by avoiding the need for a separate reverse transcription step,^27^ which is inefficient at low concentrations of TNA.^28^ Relative to prior work,^4,29^ a photocleavable linker was included in the region connecting the dsDNA to the TNA sequence to promote efficient DNA recovery and amplification by droplet PCR (Figures 1d and S3). The libraries were designed at the DNA level to contain a 30 nucleotide (nt) random region (theoretical diversity ∼10^18^ unique sequences) that was flanked on both sides with fixed-sequence primer binding sites (PBS) and a unique bar code positioned between the 5′ PBS and random region (Tables S1 and S2). Each linear single-stranded DNA library was ligated at the 3′ end to a DNA stem-loop structure that served as the primer for TNA synthesis on the DNA template (Figure 1c). Extension of the primer using a TNA polymerase^30^ and chemically synthesized TNA triphosphates (tNTPs)^25^ (Figure 1b) afforded a long hairpin with the DNA template base paired to its complementary TNA strand.^27^ The TNA region of the duplex was displaced in a separate step by extending a DNA primer annealed to the loop region of the hairpin with DNA using Bst DNA polymerase, a family-A DNA polymerase isolated from the bacterial species *Geobacillus stearothermophilus* that functions with strong strand displacement activity.^31^ The product of strand displacement is a set of six chemotype libraries prepared in a DNA display format that allows for a genotype–phenotype linkage between the DNA and TNA portions of the molecule (Figure 1c).

An activity screen was performed in parallel to evaluate the propensity for the various function-enhancing side chains (i.e., Leu, cP, Phe, Dx, and Trp) to increase the abundance of TNA aptamers present in unbiased pools of random sequences. Guided by the results of the binding assay, the screen was performed with condition 15 [10 mM HEPES (pH 7), 150 mM NaCl, 3 mM EDTA, and 0.05% Tween 20], which included 0.5 mg/mL BSA and 0.05 mg/mL ssDNA as general protein and DNA competitors, respectively. Similar to the binding assay (Figure 1d), each chemotype library (10^12^ molecules) was passed through a negative selection step to remove sequences with an affinity for Ni-NTA beads and a positive selection step for binding to the S1-RBD protein. Sequences that bound to the protein were partitioned away from the unbound material by passing the mixture over Ni-NTA beads to capture the aptamer–protein complexes on a solid-support matrix. The beads were washed 3 times with selection binding buffer, once with 2 M urea, and twice with PCR buffer [10 mM Tris-HCl, 50 mM KCl pH (8.3)]. The double-stranded DNA region encoding the bound TNA molecules was photocleaved and amplified by droplet PCR to ensure even amplification of the eluted material. The resulting amplicons were further amplified in solution by bulk PCR and submitted for HTS.

**Figure 3:**
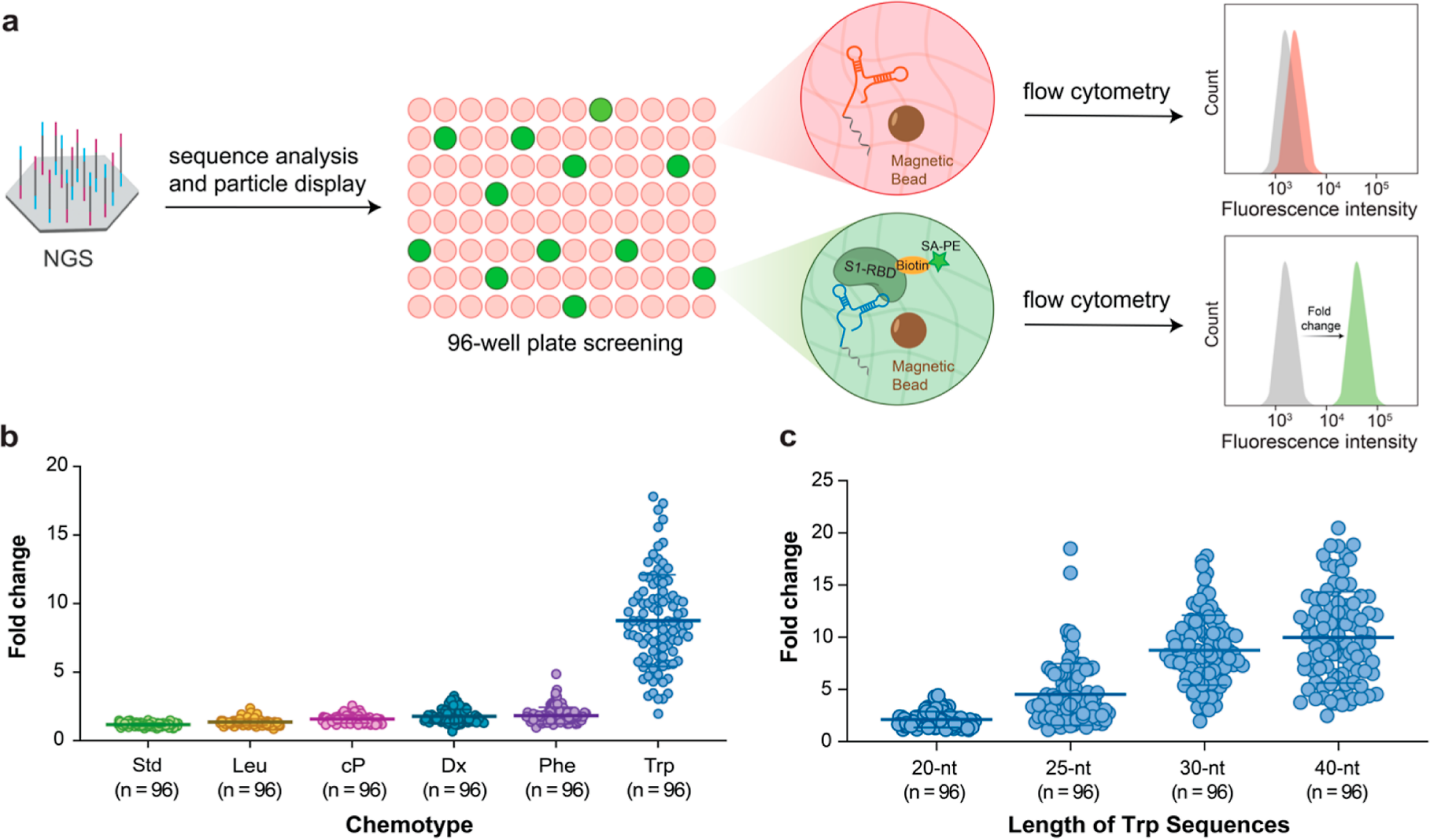
Secondary screening. (a) Cartoon representation of a flow cytometry assay performed using TNA aptamer hydrogel particles prepared by primer extension and template stripping in a hydrogel particle format. Sequences with affinity to a biotinylated S1-RBD fluoresce when incubated with PE-labeled SA. The binding activity level of sequences isolated from diverse chemotype pools is assessed in 96-well format by flow cytometry. (b) Flow cytometry analysis of TNA aptamers isolated from diverse chemotype libraries. (c) Flow cytometry analysis of TNA aptamers isolated from Trp libraries with random regions of varying lengths. All assays were performed in 96-well format in binding buffer (10 mM HEPES at pH 7, 150 mM NaCl, 3 mM EDTA, and 0.05% Tween 20). Fold change represents the average fluorescence observed from sampling 100,000 particles per sequence. Sample size (*n* = 96 sequences) per chemotype and/or library length. Abbreviations: standard (std), leucine (Leu), cyclopropyl (cP), phenylalanine (Phe), dioxol (Dx), and Trp.

To illuminate the relationship between library chemotype and function, 96 representative members from each chemotype family were evaluated for binding to S1-RBD using a highly parallel hydrogel aptamer particle strategy.^32^ In contrast to traditional particle display,^33^ whereby DNA aptamers are attached directly to the surface of a magnetic particle using an emulsion PCR and template stripping protocol, hydrogel aptamer particles are prepared by extension of a DNA primer that has been cross-linked into the gel matrix of a polyacrylamide hydrogel shell encapsulating a magnetic particle.^32^ The TNA aptamer is generated by primer extension using a DNA template encoding the desired TNA sequence. This microfluidics-free approach, which allows reagents and enzymes to pass freely in and out of the matrix, is necessary because a polymerase has not yet been discovered that can amplify XNAs by PCR. Following TNA synthesis, the template is removed and the TNA aptamers are folded by heating and cooling the hydrogel particles in binding buffer [10 mM HEPES pH 7, 150 mM NaCl, 3 mM EDTA, and 0.05% Tween 20]. The resulting monoclonal TNA aptamer hydrogel particles are evaluated for binding to a biotin-modified version of S1-RBD in 96-well format using an ELISA-type assay that is compatible with flow cytometry (Figure 3a). In this assay, aptamers with affinity to S1-RBD fluoresce at levels indicative of their binding affinity when incubated with phycoerythrin (PE)-labeled streptavidin (SA).^32^ Consequently, aptamers exhibiting high mean fluorescence values relative to aptamer-deficient hydrogel particles (DNA primer only), viewed here as a background control, are predicted to function with a higher target binding affinity due to their ability to remain bound to the target protein following iterative wash steps. This approach dramatically accelerates the pace of XNA aptamer characterization by reducing the scale of aptamer synthesis and eliminating the need to individually purify each TNA sequence by denaturing polyacrylamide gel electrophoresis.

Flow cytometry analysis of the six chemotype families (576 sequences) offered striking insights into the potential for function-enhancing side chains to increase the abundance of TNA aptamers in a random-sequence library. In an effort to minimize bias, we standardized the sequence selection criteria across aptamer binding experiments by randomly choosing sequences that contained 30% chemical modifications (or T for the std chemotype) and no more than three identical nucleotides in a row from the NGS data collected from the elution fractions of each chemotype library screen. Other levels of chemical modifications were not evaluated in this study. The resulting flow cytometry data indicate (Figure 3b) that the Trp side chain yields the highest gain-of-function activity with an average fold change of ∼9 for the population relative to background measurements observed for aptamer-deficient particles. By comparison, the other four chemotypes (Leu, cP, Phe, and Dx) function with activity levels similar to the standard base chemotype, which carries only natural bases (∼2-fold over background). This profile, which may be specific to S1-RBD, is consistent with the tendency for Trp side chains to increase the success rate of DNA aptamers.^34^ Coincidentally, the Trp side chain is also commonly found on the paratope of protein–antibody cocrystal structures,^35^ implying that our design converged on a solution that mimics nature’s approach to protein binding affinity reagents.

Inspired by the enhanced functional activity exhibited by the Trp-modified TNA library, we wished to gain a deeper understanding of the length dependency of the random region on the discovery rate of functional threomers. We were particularly interested in the question of whether certain highly active chemotypes could promote the discovery of shorter-length threomers. If so, such an approach would offer a viable route to threomers that could eventually be produced, resulting in higher yields and lower costs than equivalent reagents of longer lengths. To explore this question, we screened three new Trp chemotype libraries with random regions of 20, 25, and 40 unbiased nucleotides for binding to S1-RBD. Flow cytometry analysis of 96 representative members from each Trp chemotype library (384 sequences) revealed a positive correlation between library length and activity (Figure 3c), consistent with the theory that longer sequences have a greater propensity to fold into active structures.^15^ However, it was interesting to note that two of the threomers isolated from the 25 nt library exhibited binding response levels that are similar in magnitude (18-fold) to those of the best 30 and 40 nt threomers, indicating that shorter Trp-modified aptamers, though more rare, are still capable of achieving high-affinity binding to S1-RBD.

**Figure 4:**
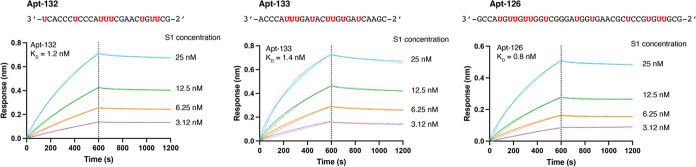
Kinetic binding analysis of Trp-modified threomers to S1. Background subtracted BLI sensorgrams comparing threomers Apt-132 (left), Apt-133 (middle), and Apt-126 (right). Curve fitting was performed with a 1:1 binding model. Binding assays were measured in binding buffer [10 mM HEPES (pH 7), 150 mM NaCl, 3 mM EDTA, and 0.05% Tween 20]. Kinetic values provided in Table S3. Trp-modified residues in the sequence are indicated in red. Threomers were enzymatically prepared using a 5′ DNA primer (not shown).

To quantify the binding properties of the hits identified by flow cytometry, we measured full kinetic titration curves by biolayer interferometry (BLI) for the top 17 threomers ranked according to their observed fold change in our ELISA-based flow cytometry assay. Each aptamer was prepared by primer extension using a 5′ biotinylated DNA primer and complementary DNA template (Table S2). The resulting aptamers were PAGE purified, electroeluted, and UV quantified. Aptamers were immobilized on SA-coated biosensor tips and evaluated for binding to the S1 protein. Curve fitting reveals that the data conform to a 1:1 binding model with calculated dissociation constants (*K*_D_) spanning a narrow range of 0.8–3.7 nM (Table S3). Close inspection of the binding curves (Figure S1) demonstrates that the 17 threomers function with off-rates (*k*_off_ 1.3–8.8 × 10^–4^ s^–1^) that are comparable to high-quality antibodies.^36,37^ Interestingly, the two top performing 25 nt threomers ranked second and third out of the 17 threomers evaluated by BLI (*K*_D_ = 1.4 nM and *K*_D_ = 1.2 nM, respectively) and were only ∼2-fold weaker in activity than the top threomer (*K*_D_ = 0.8 nM) identified in our screen (Figure 4). Importantly, these values are comparable to S1 binding aptamers generated by more conventional selection protocols involving multiple rounds of selection,^38,39^ indicating that high-throughput parallel screening can identify high-affinity aptamers without the need for iterative rounds of Darwinian evolution.

Recognizing the Trp side chain as a critical factor responsible for achieving high-affinity binding, we decided to perform a structure–activity relationship (SAR) study to confirm that the S1-RBD binding activity was dependent on the presence of the Trp moiety. Flow cytometry analysis performed on the top 17 S1-RBD binding threomers identified by hydrogel particle screening (Figure 3c), each prepared as hydrogel particles with std, Phe, and Trp chemotypes, exhibits a clear dependency of binding (*p* < 0.001) on the Trp chemotype for S1-RBD binding activity (Figure S2). The data show that high-activity binding is abrogated when the Trp side chain is replaced with a methyl group from the analogous thymine base found in the std library or a uracil residue carrying Phe at the C-5 position.

**Figure 5:**
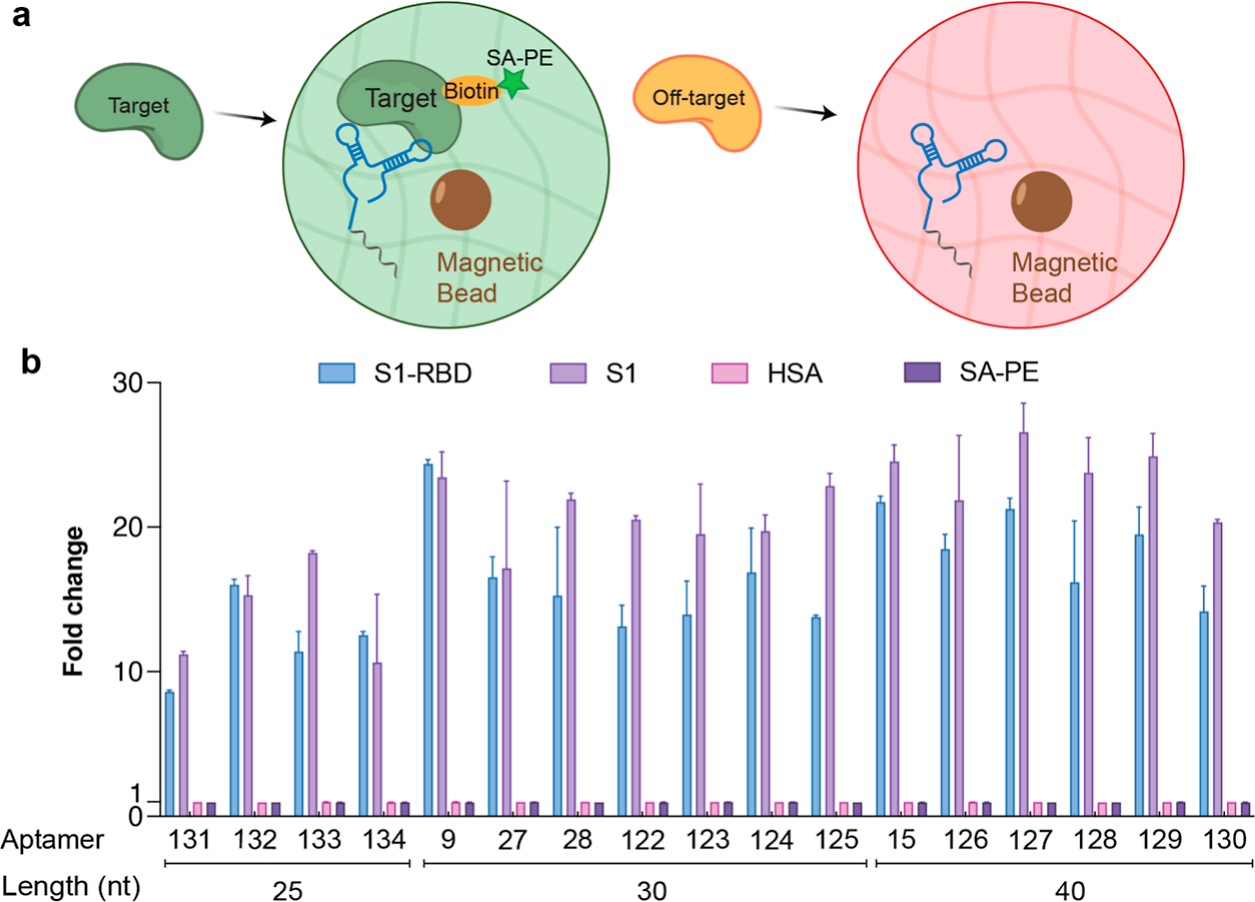
Aptamer specificity. (a) Cartoon representation of a target specificity assay performed using TNA aptamer hydrogel particles. Sequences with affinity to their cognate biotin-modified protein fluoresce when incubated with PE-labeled SA. Sequences with low off-target binding yield low fluorescence. (b) Flow cytometry analysis of Trp-modified TNA aptamers selected to bind the receptor binding domain of the S1 viral coat glycoprotein (S1-RBD) of SARS-CoV-2. Each aptamer sequence was evaluated for on-target binding to S1-RBD and S1 and for off-target binding to HSA and SA. Error bars denote ±standard deviation of the mean for 2 independent replicates. Two-tailed Student’s *t*-test reveals a statistically significant difference (*p* ≤ 0.001) between on-target and off-target values for all samples tested. All reactions were performed in binding buffer (10 mM HEPES pH 7, 150 mM NaCl, 3 mM EDTA, and 0.05% Tween 20).

Concerned that threomers obtained from a high-throughput screen might function with general protein-binding activity, we chose to examine the target binding specificity of the top 17 S1-RBD binding threomers in the particle display format. In separate flow cytometry assays, the aptamer hydrogel particles were challenged to distinguish their cognate S1-RBD target as well as the full-length S1 protein containing the RBD domain from two off-target proteins, which included human serum albumin (HSA) and SA (Figure 5a). HSA and SA were chosen based on their tendency to recognize nucleic acid sequences via nonspecific binding modes. The resulting data demonstrate that the threomers, at least in the context of this assay, exhibit high specificity for S1-RBD and S1 and show no appreciable activity for the off-target proteins (Figure 5b). The average fold change over background for the S1-RBD and S1 targets ranged from ∼10 to ∼25, indicating strong aptamer–target binding in the hydrogel matrix. By comparison, identical experiments performed with HSA and SA (Figure 5b) resulted in binding levels that were at or near background. Together, these data provide convincing evidence that high-throughput library screening can yield threomers that function with both high affinity and high specificity.

## Discussion

Aptamers are typically discovered through an iterative process of Darwinian evolution in which many recursive cycles of in vitro selection and amplification are performed to identify individual sequences that can bind to a desired target with high affinity.^15^ Top-performing sequences are then refined by directed evolution or via a post-SELEX chemical optimization process in which additional chemistry is used to enhance the physicochemical properties of the molecule.^40,41^ This was the approach taken to discover Pegaptanib (Macugen), the first therapeutic aptamer to receive approval by the US Food and Drug Administration (FDA) for the treatment of age-related macular degeneration.^42^ However, the inherently slow nature of this methodology, which can take several months to complete, severely restricts the pace of aptamer discovery. While automated protocols have been established to streamline the discovery of DNA and RNA aptamers,^43^ such approaches require specialized equipment and follow procedures that are not readily transferrable to XNAs due to a lack of polymerases that can amplify XNA by PCR. Although machine learning algorithms provide an opportunity to address this problem,^14^ reinforcement learning necessitates large amounts of data that are not presently available for alternative genetic systems, like TNA, that are only beginning to emerge as putative genetic systems for therapeutic aptamers.^44^

The current study describes a new approach to aptamer discovery that bypasses the Darwinian evolution process by using a library screening strategy that relies on the presence of function-enhancing side chains to increase the abundance of functional aptamers in unbiased pools of random sequences. We demonstrated the effectiveness of this approach by identifying threomers from a single round of high-throughput screening that bind the S1-RBD protein with binding affinities values (low to subnanomolar) comparable to monoclonal antibodies^36,37^ and aptamers evolved from conventional in vitro selection protocols.^38,39^ SAR studies confirmed the importance of the function-enhancing side chain, a Trp chemotype, as a critical element for target binding affinity and specificity. Functional analysis of hundreds of TNA sequences revealed that the Trp side chain is able to augment the TNA scaffold with physicochemical properties that were not observed in screens performed against other chemotype categories. This example is qualitatively similar to the complementary determining region of antibodies, which use large aromatic residues to shape the paratope surface with hydrophobic interactions that help drive epitope binding. However, unlike antibodies, threomers are conceptually much easier to discover as targets can be queried entirely by in vitro means that avoid the use of animal models and complex cellular expression systems that are prone to contamination and difficult to scale.

Collectively, these findings represent a possible paradigm shift in the way that aptamers are discovered. Rather than following the traditional model of one target, one library, and many rounds of selection, researchers now have an opportunity to investigate a range of targets and library designs in parallel and under experimental conditions that minimize the problem of amplification bias that arises when multiple rounds of selection are performed against the same target.^45^ As these studies continue, it will be interesting to see how their results shape our understanding of such fundamental questions as how reproducible evolutionary trajectories are in sequence space and what underlying chemical forces enable chemotypes to enhance biopolymer function. In the area of biotechnology, highly parallelizable screening platforms, such as the one described herein, could fast-track the rise of therapeutic aptamers by drastically lowering the barrier required for reagent discovery. The data generated from these experiments could accelerate the development of computational tools that can identify aptamers de novo or predict sequences that function with even greater activity than what might be observed in an experimental dataset. Since TNA is invisible to biological nucleases, the resulting reagents can be used directly without the need for the medicinal chemistry steps required to stabilize the backbone structure of DNA and RNA aptamers.

## Conclusions

We demonstrate that high-throughput screening of functionally enhanced TNA libraries offers a viable route for discovering threomers that can bind their targets with high affinity and high specificity. We suggest that the ability to bypass conventional in vitro selection protocols could help narrow the gap between aptamers and antibodies by providing a highly parallelizable path for querying diverse chemical repertoires and may offer a viable route for accelerating the discovery of therapeutic aptamers.

## Supporting Information Available

Sequence information for the DNA libraries; DNA oligonucleotides; BLI analysis for threomers; qPCR data; thermal stability of a 76-nucleotide region; schematic of the DNA display strategy; BLI sensograms; and structure–activity analysis (PDF)
