# Supplementary material for: Functionally Enhanced XNA Aptamers Discovered by Parallelized Library Screening
Source: J Am Chem Soc. 2023 Nov 14;145(47):25789–96. doi: 10.1021/jacs.3c09497 (PMC10690791; doi:10.1021/jacs.3c09497)
Supplement: Supplementary file 1 — ja3c09497_si_001.pdf [file ja3c09497_si_001.pdf]

# Supplementary Information

# Functionally Enhanced XNA Aptamers Discovered by Parallelized Library Screening

Adriana Lozoya-Colinas[1⊥], Yutong Yu[1⊥], and John C. Chaput[1-4*]

[1]Department of Pharmaceutical Sciences, University of California, Irvine, CA 92697-3958, United States
[2]Department of Chemistry, University of California, Irvine, CA 92697-3958, United States
[3]Department of Molecular Biology and Biochemistry, University of California, Irvine, CA 92697-3958, United States
[4]Department of Chemical and Biomolecular Engineering, University of California, Irvine, CA 92697-3958, United States

[⊥] These authors contributed equally

**Table of Contents:**

**Materials and Methods**

**Supplementary Tables**
- Table S1. Sequence information for the DNA libraries
- Table S2. DNA oligonucleotides used in the study
- Table S3. Summary of BLI analysis for threomers binding to the S1 protein

**Supplementary Figures**
- Figure S1. Example of qPCR data used to evaluate the selection conditions
- Figure S2. Thermal stability of a 76-nucleotide region of the double-stranded DNA used in the DNA display format
- Figure S3. Schematic of the DNA display strategy
- Figure S4. BLI sensorgrams for the top 17 S1-RBD binding threomers
- Figure S5. Structure-activity analysis of top performing threomers

## Materials and Methods

**General information**. T4 DNA ligase buffer, ThermoPol buffer, Mg-free Taq polymerase buffer, and Bst 3.0 DNA polymerase were purchased from New England Biolabs. PCRBIO HiFi polymerase was purchased from PCRBiosystems. TNA polymerase and T7 DNA ligase were expressed and purified as described previously.[1] DNA triphosphates were purchased from Thermo Fisher Scientific. TNA triphosphates were synthesized as described previously.[2-4] DNA oligonucleotides were purchased from Integrated DNA Technologies. DNA libraries were purchased from the Keck Oligonucleotide Synthesis Facility at Yale. For the aptamer selection, Ni-NTA agarose resin was purchased from Qiagen. His-tagged SARS-CoV-2 spike glycoprotein subunit 1 (S1) and SARS-CoV-2 receptor binding domain (S1-RBD) of the spike glycoprotein subunit 1 were purchased from ACRO Biosystems. Bovine serum albumin (BSA) and salmon sperm DNA were purchased from Thermo Fisher Scientific. Human $\alpha$-thrombin was from Haematologic Technologies. Lyophilized powder of albumin from human serum (HSA) was bought from Sigma-Aldrich. Phenol:chloroform:isoamyl alcohol 25:24:1 (saturated with 10 mM Tris, pH 8.0, 1 mM EDTA) was purchased from Sigma-Aldrich. Amicon™ ultra centricon centrifugal filter units were purchased from EMD Millipore. DNA clean & concentrator-5 kit was from Zymo Research. For the BLI assay, streptavidin biosensors were purchased from ForteBio. For particle display, magnetic beads (Dynabeads™ M-270 Carboxylic Acid) and magnets (DynaMag-96 Side Skirted and DynaMag-2) were purchased from Thermo Fisher Scientific. Ammonium persulfate was purchased from Sigma-Aldrich. EZ-Link™ NHS-PEG4-Biotin and streptavidin-phycoerythrin (SA-PE) were purchased from Thermo Fisher Scientific.

**Library synthesis**. TNA libraries were designed at the DNA level to contain unbiased internal variable regions of 20, 25, 30, or 40 nucleotides (25% of A, T, G, and C) flanked on both sides by fixed-sequence primer binding sites. Each library contained a 6 nt barcode located between the forward primer and random region. Library preparation followed the same protocol as described previously[5] with a few modifications. The phosphorylated DNA hairpin primer containing a photocleavable linker was ligated to the library by combining 0.5 nmol of DNA library with 0.6 nmol of DNA hairpin in a final volume of 100 □L of 1× T4 DNA ligase buffer. The libraries were annealed to the hairpin by heating for 5 min at 95°C and cooling for 5 min at 4°C. T7 DNA ligase was then added to a final concentration of 0.5 □M and the reaction was incubated overnight at 24°C. The resulting self-priming DNA libraries were purified by 10% denaturing polyacrylamide gel electrophoresis (PAGE), electroeluted, and quantified by $A_{260}$ absorbance with the Nanodrop spectrophotometer.

Each self-priming DNA library (100 pmol) was extended with tNTPs to form a chimeric TNA/DNA heteroduplex. A reaction solution with a final concentration of 1 □M of hairpin library and 1× ThermoPol buffer was heated for 5 min at 95°C and cooled for 5 min at 4°C. TNA polymerization was initiated by adding the TNA polymerase (1 µM final concentration) and 100 µM of each tNTP (tATP/tCTP/tGTP/tTTP or C-5 modified tUTP), the solution was incubated for 2 h at 55°C. Afterwards, excess polymerase was extracted by adding an equal volume of phenol/chloroform/isoamyl alcohol (25:24:1, saturated with 10 mM Tris, pH 8.0, 1 mM EDTA) and mixing by inversion until cloudy, followed by centrifugation at 12,000 rpm for 5 min. Following extraction, the top layer was collected and the remaining tNTPs were removed by exchanging into water using a YM-30 spin filter.

The strand displacement step was performed by extending a DNA primer annealed to the hairpin loop with dNTPs. Accordingly, the library (1 □M as the final concentration) was resuspended in 1× ThermoPol buffer containing 500 □M dNTPs and 2 □M strand displacement primer. After heating for 5 min at 95°C and slowly cooling to room temperature, the displacement reaction was initiated by adding Bst 3.0 DNA polymerase to a final concentration of 80 U/mL. The reaction was incubated for 1 h at 50°C. Bst polymerase was extracted with phenol/chloroform/isoamyl alcohol as described above. The TNA library displayed on dsDNA was separated from the residual phenol and reaction components in a YM-30 spin filter. Any remaining phenol was removed by adding 900 □L of ethanol to the solution and drying completely with the SpeedVac vacuum concentrators. The libraries were resuspended in 400 □L of the binding buffer (10 mM HEPES pH 7, 150 mM NaCl, 3 mM EDTA, 0.05% Tween 20, 0.5 mg/mL of BSA and 0.05 mg/mL of salmon sperm DNA).

**Optimization of the binding and wash conditions.** Aptamer selections were performed by incubating library L24.1 prepared with the Phe chemotype (10 pmol, ~$10^{12}$ unique sequences) with Ni-NTA agarose resin for 30 min at 24°C with rotation in one of two different selection buffers: buffer 1 contained 25 mM Tris pH 8.0, 150

mM NaCl; and buffer 2 contained 10 mM HEPES pH 7, 150 mM NaCl, 3 mM EDTA, 0.05% Tween 20, 0.5 mg/mL BSA, 0.05 mg/mL salmon sperm DNA. The material collected from the flow-through of the negative selection (Ni-NTA agarose resin only) was incubated with the His-tagged S1 poised at a 100:1, 10:1 or 1:1 (library:target) ratio for 15 min at 24°C with rotation. The aptamer/S1 solution was then added to a column with Ni-NTA agarose resin and incubated for 15 min at 24°C with rotation. The column was drained and washed 3 times with 400 □L of selection buffer to remove unbound and weakly bound molecules. A stringent wash step was performed using buffer supplemented with a) 1 M NaCl, b) 0.5 M urea, c) 1 M urea, d) 2 M urea, e) no additive. The column was then washed twice with Mg-free Taq polymerase buffer [10 mM Tris-HCl, 50 mM KCl pH (8.3)]. The column was exposed to UV radiation for 1 hour (UVP crosslinker CL-1000L, 365 nm) to release the encoding dsDNA from the S1-RBD bound Ni-NTA agarose resin. Conditions that included an aptamer refolding step involved heating the solution to 65°C for 5 min and cooling on ice for 15 min prior to the initial negative selection step.

**qPCR analysis.** The starting DNA library and eluted material recovered after photocleavage were evaluated by qPCR to assess the stringency of each binding and wash condition. For each measurement, a linear calibration plot was obtained by performing a qPCR analysis on dilution samples of the starting ssDNA library (L24.1, Table S1) that spanned a concentration with 5-fold dilutions from 10 nM to 3.2 pM. The analyzed samples correspond to the DNA display library before selection and after elution via photocleavage (see above). The DNA display library and eluted material were diluted such that their final concentration for qPCR occurred within the linear range of the calibration plot. Reported values were the average of 3 experimental replicates. An example of the qPCR data and analysis can be found on Supplementary Figure S1.

**Aptamer selection.** Aptamer selections were performed by first refolding the library (100 pmol, ~$10^{12}$ unique sequences) as described above in binding buffer 2 (10 mM HEPES pH 7, 150 mM NaCl, 3 mM EDTA, 0.05% Tween 20, 0.5 mg/mL BSA, 0.05 mg/mL salmon sperm DNA) and incubating with Ni-NTA agarose resin for 30 min at 24°C with rotation. The material collected from the flow-through of the negative selection (Ni-NTA agarose resin only) was incubated with the His-tagged S1-RBD poised at a 10:1 (library:target) ratio for 15 min at 24°C with rotation. The aptamer/S1-RBD solution was then added to a column with Ni-NTA agarose resin and incubated for 15 min at 24°C with rotation. The column was drained and washed 3 times with 400 □L of selection buffer to remove the unbound and weakly bound molecules. A stringent wash step was performed with 2 M urea to reduce the occurrence of nonspecific binding followed by 2 washes with Mg-free Taq polymerase buffer (10 mM Tris-HCl, 50 mM KCl pH 8.3). The column was exposed to UV radiation for 1 hour (UVP crosslinker CL-1000L, 365 nm) to release the encoding dsDNA from the S1-RBD bound Ni-NTA agarose resin. The recovered DNA was amplified first by droplet PCR to minimize sequence bias, followed by a regular bulk PCR with library-specific PCR primers and purified using DNA Clean and Concentrator columns from Zymo Research.

**High Throughput Sequencing (HTS)**. The recovered amplicons were prepared as barcoded libraries and sequenced on a NovaSeq6000 with the S4 cell with the paired end setting for 100 cycles for an estimated 200 M reads per library. Sequencing data was then filtered, trimmed, and aligned. Among the filtering conditions were full-length reads on both directions and ≤4 consecutive bases. The sequences were organized based on their percent T content for the std library, and C-5 modified U for the functionally-enhanced libraries.

**Hydrogel bead preparation**. Hydrogel particles were prepared as previously described.[6] In brief, $8\times10^8$ carboxylic magnetic beads were transferred to a 5 mL Eppendorf tube. After removal of the supernatant by placing the tube in the magnet, 1056 uL of 6% degassed acrylamide:bisacrylamide (19:1), 120 □L of 1 mM acrydite-modified primer PBS8 and 24 □L of freshly made 30% (w/v) ammonium persulfate (APS) were added and the beads were carefully mixed with the pipette without introducing any air to the mixture. Then 3.6 mL of previously degassed oil mixture (4%/20%/76%; w/w/w; KF6036 / mineral oil / DMF H6CS) were added to the top of the acrylamide solution without mixing and the top oil layer was degassed again for 10 min. Then 4 □L of TEMED were added to the oil mixture and the headspace of the tube was purged for another 60 seconds. The tube was placed on a Benchmark Bead bug-6 for 65 s at 2500 rpm and then placed on ice to polymerize for 4 hours. The beads were then washed with breaking buffer (10 mM Tris-HCl pH 7.5, 100 mM NaCl, 1 mM EDTA, 1% (v/v) SDS, 1% (v/v) Triton x100) until no oil was observed. The beads were resuspended in 4 mL of breaking buffer and counted on a cell counter hemocytometer.

**Transcription in hydrogel beads**. Aptamer hydrogel particles were prepared as previously described[6]. In brief, 3 million of 5' acrydite-modified PBS8 polymerized hydrogel-coated beads were transferred to each well of a 96-well plate and washed twice with 100 □L of 1x Thermopol buffer (20 mM Tris-HCl, 10 mM $(NH_4)_2SO_4$, 10 mM KCl, 2 mM $MgSO_4$, 0.1% Triton® X-100, pH 8.8@25°). Then a reaction mixture with a final volume of 25 □L was prepared in each well with 1×Thermopol buffer, 2 □M of template, 0.01% of KF6012, and water. The plate was heated at 90°C for 3 minutes and then cooled down to 4°C for 5 minutes to anneal. TNA polymerization was initiated by adding 100 □M of each tNTP and 2 □M of TNA polymerase and the reaction was incubated at 55°C for 4 hours. Once the reaction was completed, the beads were washed twice with breaking buffer and resuspended for a final concentration of 25,000 beads per □L.

**Screening of hydrogel beads**. Approximately 500,000 hydrogel beads with transcribed TNA products were transferred to a new plate. The same amount of primer-polymerized only hydrogel beads was set as the negative control. The template strand was removed by adding 100 mM NaOH containing 0.5% Tween 20 and incubated for 3 minutes, then adding an equal volume of breaking buffer and removing all the supernatant. The stripping step was done twice. Then the beads were washed with 100 □L of breaking buffer twice and BLI binding buffer (10 mM HEPES pH 7, 150 mM NaCl, 3 mM EDTA, 0.05% Tween 20) twice more. The plate was heated at 90°C for 3 minutes and then cooled to 4°C for 5 minutes to fold the aptamers into their active structure. RBD was biotinylated using 20 molar fold excess of EZ-Link™ NHS-PEG4-Biotin at room temperature for 30 minutes and followed by three PBS (137 mM NaCl, 2.7 mM KCl, 8 mM $Na_2HPO_4$, 2 mM $KH_2PO_4$) washes using the YM-10 Centricon spin column. Biotinylated RBD was then added to each well to a final concentration of 100 nM in 50 □L and incubated for 30 minutes at room temperature. After 30 minutes, the supernatant was removed, and beads were resuspended in 50 □L of BLI binding buffer and 0.5 □L of SA-PE was added to the solution. After 15 minutes of incubation, the supernatant was removed, the beads were resuspended in 50 □L of BLI binding buffer, and samples were run in the flow cytometer in sets of 24. Flow cytometer conditions: 600 rpm mix every 8 samples for 5 seconds, rinse after every sample, 20 □L of sample is taken, gating: SSC-H ($10^6$) x FSC-H ($10^6$) (0.4 x 0.4 for 2.8 □m Dyna beads).

**Aptamer preparation for kinetic analysis**. Aptamers identified by sequencing were prepared as biotin-labeled TNA molecules using the 5' biotinylated PBS8 20 nt DNA primer and the corresponding DNA template, which contained an additional 20 nucleotides $(AAAC)_5$ at the 3′ end to facilitate the band separation by PAGE. A 750 □L reaction volume containing 1 □M of both primer and template as well as 1× ThermoPol Buffer was heated for 5 min at 95°C and then cooled down to 4°C to anneal. TNA polymerization was initiated by adding 100 □M of each tNTP and 2 □M of 10-92 TNA polymerase and then incubated for 2 h at 55 °C. Full-length biotinylated aptamers were purified by 10% denaturing polyacrylamide gel electrophoresis for 1.5 h at a constant 16 W. TNA was recovered from the gel by electroelution at 60 V overnight and then buffer-exchanged into water and concentrated using a YM-10 Centricon centrifugal filter device. TNA concentration was quantified by measuring its absorbance at 260 nm with a NanoDrop spectrophotometer.

**BLI assays**. Biotinylated aptamers at a concentration of 50 nM were folded into their active conformation in BLI binding buffer (10 mM HEPES pH 7, 150 mM NaCl, 3 mM EDTA, 0.05% Tween 20) by heating to 95°C for 5 minutes and then cooling to 4°C for 5 minutes. Streptavidin-coated optical biosensors were previously equilibrated in BLI binding buffer for 30 min. For measuring full kinetics, the BLI run was performed with the following steps: a buffer only baseline for 60 s to equilibrate sensors, loading the aptamer for 200 s, a second buffer only baseline for 200 s, an association phase with the target protein for 600 s, and a dissociation phase for 600 s. Serial diluted target concentration ranged from 50 nM to 3.125 nM. The experiments were conducted at 30ºC. Data were analyzed using the Octet Data Analysis HT software. The buffer only baseline measurement was used to subtract the background from all samples before applying Savitzky-Golay filtering and fitting the association and dissociation curves and applying a global fit to determine $K_D$.

**Aptamer specificity test**. From the initial hydrogel bead screening with 4 lengths of the tryptophan-modified sequences, 17 TNA aptamer sequences were selected, which displayed the highest fold-change values with respect to the beads that contained only the primer. These 17 aptamers were synthesized on the PBS8-polymerized hydrogel beads using the method described above. Each specific synthesis was split into 4 different wells (with approximately 500,000 beads per well). The 96-well plate was heated at 90°C for 3 minutes

and then cooled to 4°C for 5 minutes to promote the folding of the aptamers. Then, 100 □M of biotinylated S1, S1-RBD, HSA, or streptavidin was added to each well and incubated for 30 min respectively. After removal of the supernatant, samples were incubated in 50 uL of BLI binding buffer with 0.5 □L of SA-PE for 15 min. The supernatant was removed, and the beads were resuspended in 50 □L of BLI binding buffer, and samples were run in the flow cytometer. The experiment was performed twice independently.

**Chemotype swap.** Hydrogel aptamer particles were separately prepared for the top 17 S1-RBD binding threomers with the std, Phe, and Trp chemotypes. Each of the three aptamer sets were prepared using the appropriate set of TNA triphosphates. Following synthesis and template stripping, the sequences were refolded by heating at 90 °C for 3 minutes and then cooled to 4°C for 5 minutes. The samples were then incubated with 100 □M of biotinylated S1 for 30 min, followed by a 15 min incubation with 0.5 □L of SA-PE. The supernatant was removed, and the beads were resuspended in 50 □L of BLI binding buffer. Lastly, the samples were run in the flow cytometer. The experiment was performed twice independently to verify its reproducibility.

**Thermal stability measurements**

Melts were performed with 1:1 oligonucleotide stoichiometry (1 □M of each strand, Table S2) in 500 mM NaCl, 10 mM sodium phosphate, 0.1 mM EDTA at pH 7 with and without 2 M urea. Prior to melting, the strands are annealed in an Eppendorf tube by heating to 95ºC for 5 minutes and cooling on ice. The heating curves were obtained in a quartz cuvette with a temperature gradient of 20º to 90ºC and a ramping rate of 1°C per min by monitoring the change in UV absorbance at 260 nm at each temperature. Tm values were either undeterminable (due to the value being outside the measured range of 20-90˚C) or determined by the maximum calculated from the first derivative of the heating curve.

**Supplementary Table S1.** Sequence information for the DNA libraries.

| Name | Sequence (5'-3')* |
|---|---|
| L24.1 | TTCTCATGTTTGACAGCTTAATTCCT-$N_9$-CTGCTTTCGCAG-$N_9$-GGTGGTATCCCCAAGGGGAC |
| L26.1 | TTCTCATGTTTGACAGCTTAAGGTGT-$N_{20}$-GGTGGTATCCCCAAGGGGAC |
| L27.1 | CTCAACCTACTACTGGGCTGCGAGGA-$N_{25}$-GGTGGTATCCCCAAGGGGAC |
| L28.1 | TAAGCGTGACCTAATATGACCGTAAG-$N_{30}$-GGTGGTATCCCCAAGGGGAC |
| L28.2 | AGTTCGCTATCGGCTGTAATAATTCA-$N_{30}$-GGTGGTATCCCCAAGGGGAC |
| L28.3 | CTGGAGCCTCAGTCCGAGTTATGTCG-$N_{30}$-GGTGGTATCCCCAAGGGGAC |
| L28.4 | CTCTGCTGAAGCCAGTTACCGAGACA-$N_{30}$-GGTGGTATCCCCAAGGGGAC |
| L28.5 | AGGGCTCTTAAAATTCGGAGGAGGTA-$N_{30}$-GGTGGTATCCCCAAGGGGAC |
| L28.6 | TTGTAGATGCTCAATACTCCCGATGG-$N_{30}$-GGTGGTATCCCCAAGGGGAC |
| L30.1 | TCGTTCTCGGACGGAGAGATATTGAA-$N_{40}$-GGTGGTATCCCCAAGGGGAC |

*Bar codes underlined.

**Supplementary Table S2.** DNA oligonucleotides used in the study.

| Name | Sequence (5'-3') |
|---|---|
| Photocleavable hairpin | /5Phos/ACTACGTACCCACAACCTCGGCCGTACCACGGTACGTAGTGTCCCCTT GGGGA/iSpPC/TACCACC |
| Strand displacement primer | GGTGGTATCCCCAAGGGGACACTACGTACCGTGGTACGGCCGAGGTTGTG |
| PBS8 | GTCCCCTTGGGGATACCACC |
| PBS 12 | TTCTCATGTTTGACAGCTTA |
| PBS 16 | CTCAACCTACTACTGGGCTG |
| PBS 20 | TAAGCGTGACCTAATATGAC |
| PBS 21 | AGTTCGCTATCGGCTGTAAT |
| PBS 22 | CTGGAGCCTCAGTCCGAGTT |
| PBS 23 | CTCTGCTGAAGCCAGTTACC |
| PBS 31 | AGGGCTCTTAAAATTCGGAG |
| PBS 32 | TTGTAGATGCTCAATACTCC |
| PBS 33 | TCGTTCTCGGACGGAGAGAT |
| Biotin-PBS8 | /5BiotinTEG/GTCCCCTTGGGGATACCACC |
| Acrydite-PBS8 | /5Acryd/GTCCCCTTGGGGATACCACC |
| Example template used to prepare the BLI samples | **CTCACAGCCTAAGGGTGCATACTGACAACG**GGTGGTATCCCCAAGGGGACAAACA AACAAACAAACAAAC (underlined, primer binding site; bold, aptamer template, plain, overhang region) |
| DNA duplex used for UV Melt study | 5'-d-TAAGCGTGACCTAATATGACCGTAAGCTCACAGCCTAAGGGTGCATACTGACAACGGGTGGTATCCCCAAGGGGAC-3'<br>3'-d-ATTCGCACTGGATTATACTGGCATTCGAGTGTCGGATTCCCACGTATGACTGTTGCCCACCATAGGGGTTCCCCTG-5' |

**Supplementary Table S3.** Summary of BLI analysis for threomers binding to the S1 protein.

| Name | TNA Aptamer sequences (3'-2') | length (nt) | target | $K_D$ (nM) | $k_{on}$ ($M^{-1}s^{-1}$) | $k_{off}$ ($s^{-1}$) | Fit ($R^2$) |
|---|---|---|---|---|---|---|---|
| 131 | ATTCGACGCGTCGTGCATATATGTG | 25 | S1 | 3.1 | $5.4 \times 10^4$ | $1.7 \times 10^{-4}$ | 0.999 |
| 132 | TCACCCTCCCATTTCGAACTGTTCG | 25 | S1 | 1.2 | $7.5 \times 10^4$ | $8.8 \times 10^{-4}$ | 0.999 |
| 133 | ACCCATTTGATACTTGTGATCAAGC | 25 | S1 | 1.4 | $1.1 \times 10^5$ | $1.6 \times 10^{-4}$ | 0.997 |
| 134 | ATGTGATTACCTGCATATGCATCCC | 25 | S1 | 3.5 | $5.5 \times 10^4$ | $2.0 \times 10^{-4}$ | 0.999 |
| 9 | CGTTGTCAGTATGCACCCTTAGGCT GTGAG | 30 | S1 | 2.0 | $8.0 \times 10^4$ | $1.6 \times 10^{-4}$ | 0.998 |
| 27 | CACGATCGATGAGCCGTTTATTGAA CTCGT | 30 | S1 | 2.4 | $6.4 \times 10^4$ | $1.5 \times 10^{-4}$ | 0.999 |
| 28 | CTGCTCAGCGTTTGCGTCTATACCG TACCC | 30 | S1 | 2.1 | $6.1 \times 10^4$ | $1.3 \times 10^{-4}$ | 0.999 |
| 122 | GTTTGGTTACCAGCGCGTCTAGTGC TAGAG | 30 | S1 | 3.7 | $6.3 \times 10^4$ | $2.3 \times 10^{-4}$ | 0.999 |
| 123 | GTCGGATGGGCTGTTACTGTACTCT CGAGG | 30 | S1 | 2.8 | $6.4 \times 10^4$ | $1.8 \times 10^{-4}$ | 0.999 |
| 124 | CTGAGGTCACTTGTAACACGCGTCC TGTCT | 30 | S1 | 3.4 | $6.8 \times 10^4$ | $2.3 \times 10^{-4}$ | 0.998 |
| 125 | CTACATTTCAATTCAGATCGCTTGG CCCGA | 30 | S1 | 2.5 | $6.9 \times 10^4$ | $1.7 \times 10^{-4}$ | 0.999 |
| 15 | TCGTTGTTGTCCTGCCTTGGCCGTA CTATGCCCCAGGCGA | 40 | S1 | 2.0 | $8.0 \times 10^4$ | $1.6 \times 10^{-4}$ | 0.998 |
| 126 | GCCATGTTGTTGGTCGGGATGGTGA ACGCTCCGTGTTGCG | 40 | S1 | 0.8 | $9.1 \times 10^4$ | $7.8 \times 10^{-4}$ | 0.999 |
| 127 | TGTTGACTCTTAGCACGGCATGCTT CCTGCGCCTTGACGA | 40 | S1 | 3.6 | $6.4 \times 10^4$ | $2.3 \times 10^{-4}$ | 0.999 |
| 128 | ATTGTCCGCTCGTCTTCGAACGCCC TGGTTACACTCCTCC | 40 | S1 | 1.8 | $7.2 \times 10^4$ | $1.3 \times 10^{-4}$ | 0.999 |
| 129 | CTGAGTATTTCTGCTAGGTGCGGCA CGCCTTGTTGCATCG | 40 | S1 | 1.8 | $7.6 \times 10^4$ | $1.4 \times 10^{-4}$ | 0.999 |
| 130 | TCGCCGCACGGATACCTCTTCATGT GTTTCCTGATACAGT | 40 | S1 | 3.3 | $4.9 \times 10^4$ | $1.6 \times 10^{-4}$ | 0.999 |

**a**

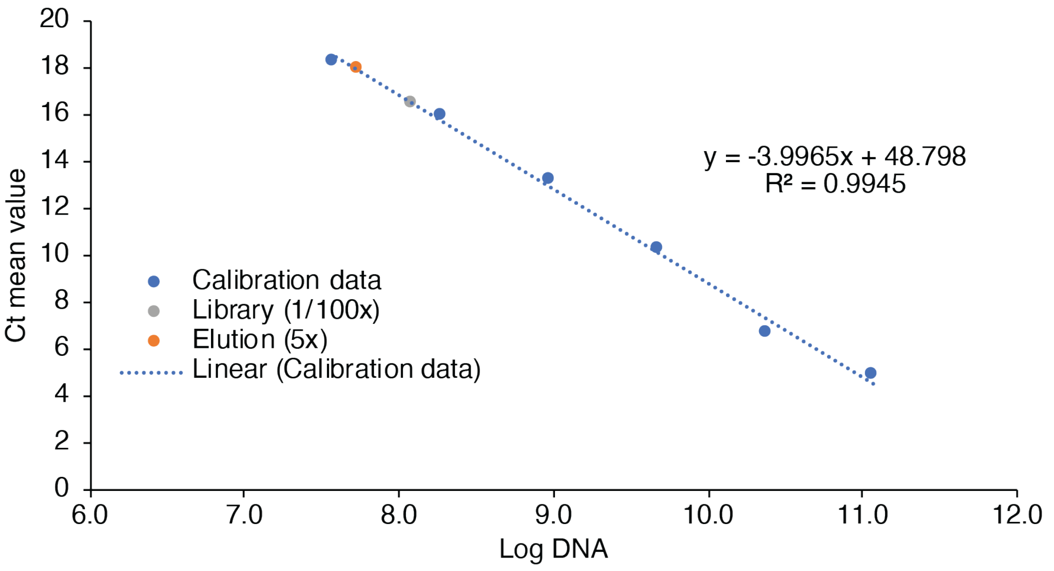


| Sample | Ct Mean | Ct SD | Log DNA | DNA | Dilution Factor | DNA | L:E |
|---|---|---|---|---|---|---|---|
| Library | 16.441 | 0.213 | 8.10 | 1.25E+08 | 40000 | 4.99E+12 | 1.84E+03 |
| Elution | 17.885 | 0.311 | 7.74 | 5.43E+07 | 50 | 2.72E+09 | |

**b**

| Concentration | CT | Ct Mean | Ct SD |
|---|---|---|---|
| 10 nM | 4.901 | 4.822 | 0.075 |
| 10 nM | 4.752 | 4.822 | 0.075 |
| 10 nM | 4.814 | 4.822 | 0.075 |
| 2 nM | 6.682 | 6.631 | 0.048 |
| 2 nM | 6.625 | 6.631 | 0.048 |
| 2 nM | 6.587 | 6.631 | 0.048 |
| 400 pM | 10.285 | 10.225 | 0.058 |
| 400 pM | 10.223 | 10.225 | 0.058 |
| 400 pM | 10.168 | 10.225 | 0.058 |
| 80 pM | 13.344 | 13.181 | 0.172 |
| 80 pM | 13.198 | 13.181 | 0.172 |
| 80 pM | 13.000 | 13.181 | 0.172 |
| 16 pM | 15.944 | 15.901 | 0.096 |
| 16 pM | 15.968 | 15.901 | 0.096 |
| 16 pM | 15.790 | 15.901 | 0.096 |
| 3.2 pM | 18.385 | 18.224 | 0.182 |
| 3.2 pM | 18.260 | 18.224 | 0.182 |
| 3.2 pM | 18.026 | 18.224 | 0.182 |

**c**

| Sample Name | CT | Ct Mean | Ct SD |
|---|---|---|---|
| Library 1/100x | 16.607 | 16.441 | 0.213 |
| Library 1/100x | 16.514 | 16.441 | 0.213 |
| Library 1/100x | 16.201 | 16.441 | 0.213 |
| Elution 5x | 18.150 | 17.885 | 0.311 |
| Elution 5x | 17.962 | 17.885 | 0.311 |
| Elution 5x | 17.543 | 17.885 | 0.311 |

**Supplementary Figure S1**. Example of qPCR data used to evaluate the selection conditions. This example illustrates the data collected for condition 13 in Figure 2b. (a) Linear calibration plot obtained by quantifying the starting library at various dilutions. Table below provides the values used to calculate the number of DNA molecules in the starting library and eluted material recovered by photocleavage. (b) Raw Ct values used to create the linear calibration plot. (c) Raw Ct values observed for the initial library and eluted material. All assays were performed in triplicate in a 96-well format.

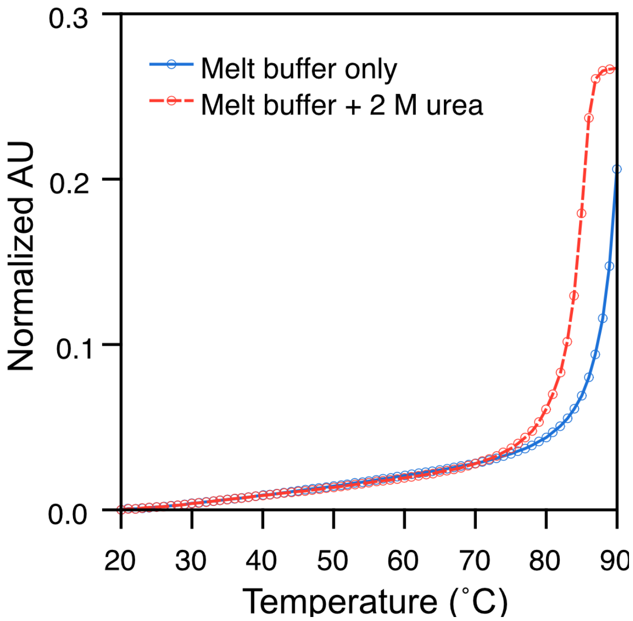


**Supplementary Figure S2**. Thermal stability of a 76-nucleotide region of the double-stranded DNA used in the DNA display format. Melts were performed with 2 μM of total oligonucleotide (1 μM of each strand, Table S2) in 500 mM NaCl, 10 mM sodium phosphate, and 0.1 mM EDTA pH 7. The Tm observed under standard conditions (solid blue) is above (>90°C) the measured temperature range of 20-90°C. The Tm of this duplex in the presence of 2 M urea (dashed red) is 85.5°C. These data confirm that the dsDNA region encoding the TNA aptamers in the DNA display format remains in the duplex form during aptamer refolding and 2 M urea wash steps.

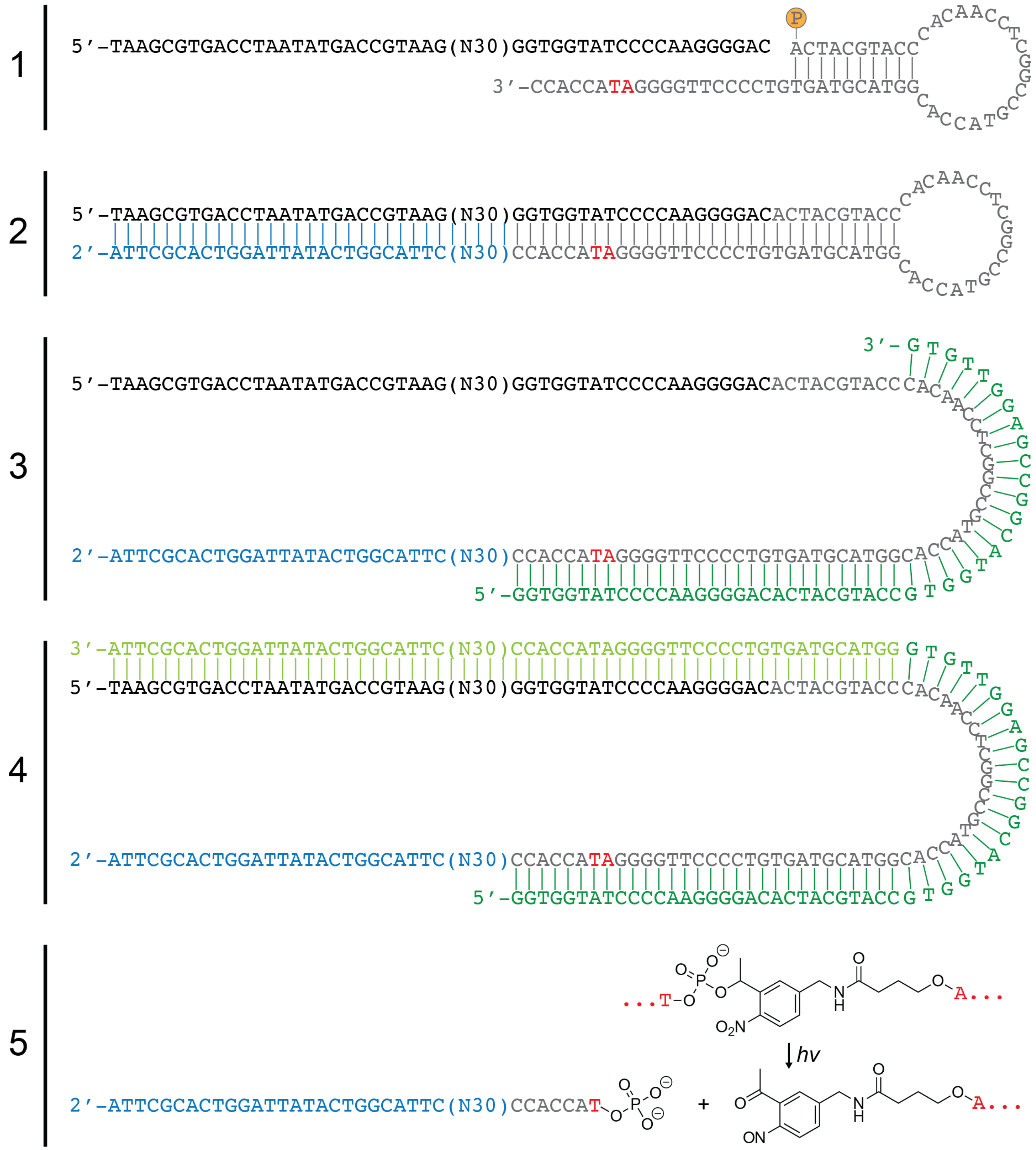


**Supplementary Figure S3**. Schematic of the DNA display strategy. Step 1: ligation of the self-priming hairpin, step 2: TNA synthesis by primer extension, step 3: annealing the strand displacement primer, step 4: extending the strand displacement primer with DNA, step 5: photocleavage to recover the encoding dsDNA.

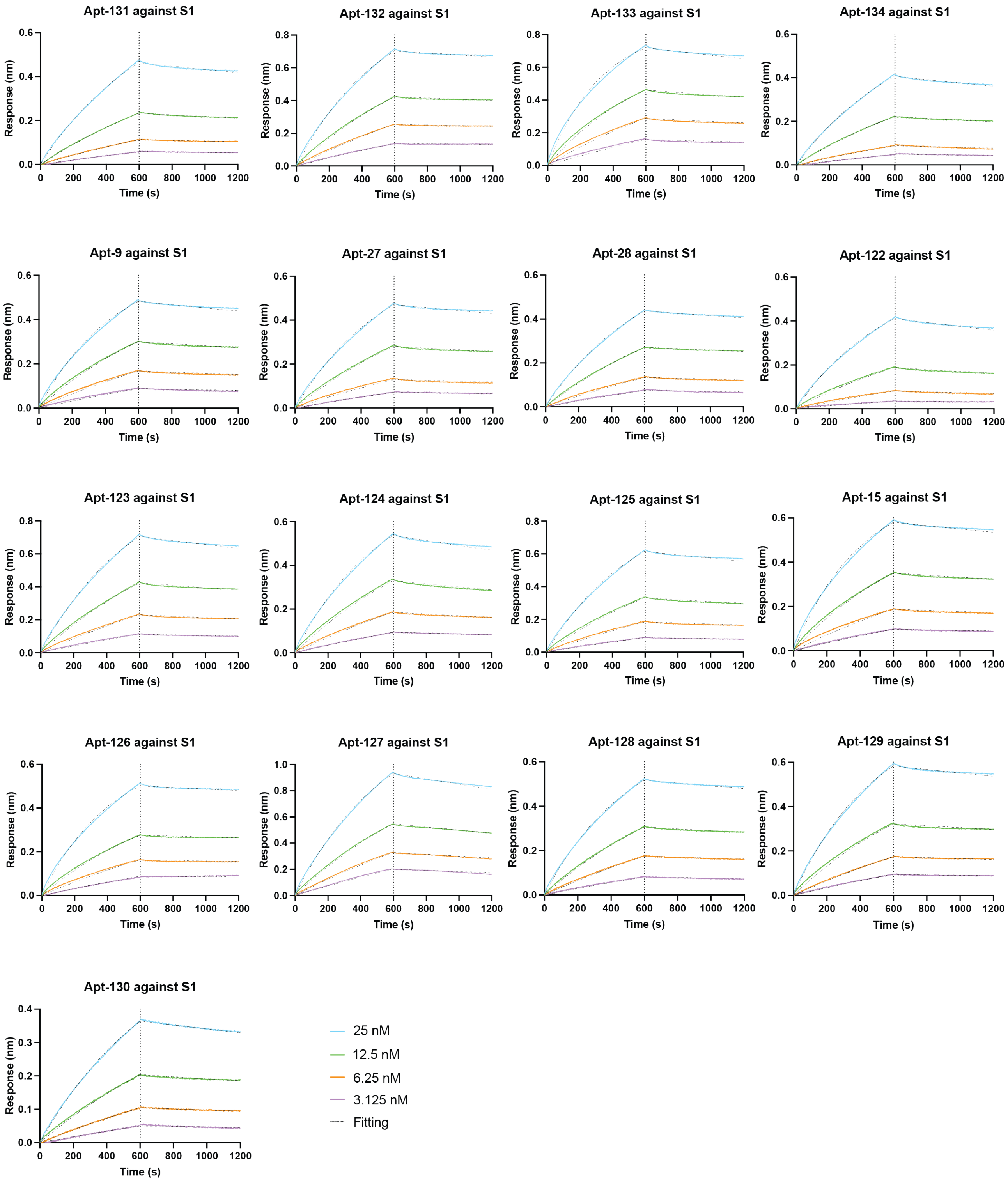


**Supplementary Figure S4**. BLI sensorgrams for the top 17 S1-RBD binding threomers. Curve fitting was performed with a 1:1 binding model using the Octet Data Analysis HT software. All assays were performed in BLI binding buffer (10 mM HEPES pH 7, 150 mM NaCl, 3 mM EDTA, 0.05% Tween 20).

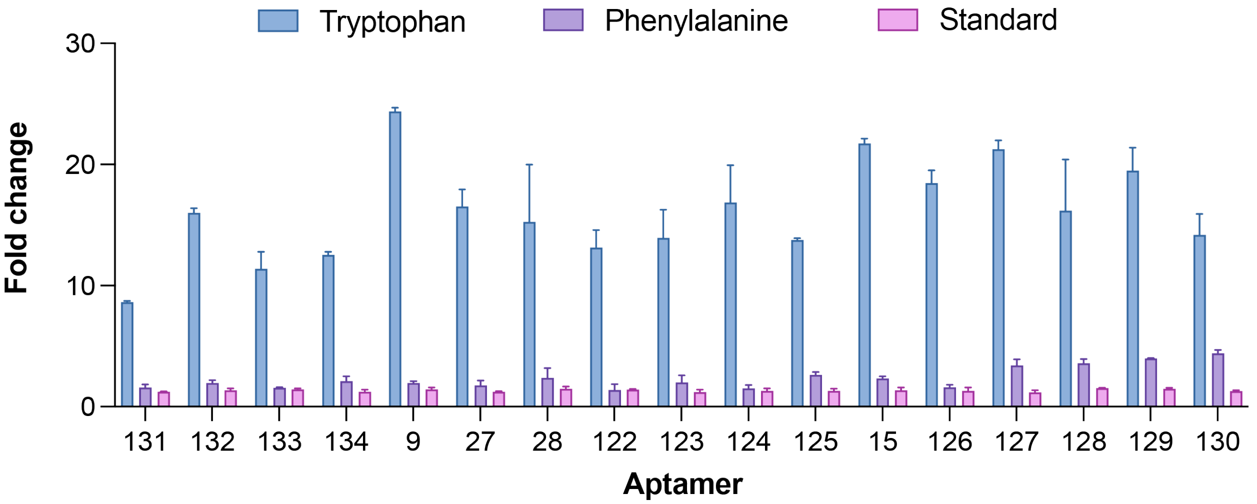


**Supplementary Figure S5**. Structure-activity analysis. The target binding specificity of the top 17 S1-RBD binding threomers were measured by flow cytometry as aptamer hydrogel particles. Error bars denote ± standard deviation of the mean for 2 independent replicates. Two-tailed Student's t-test reveals a statically significant difference ($p < 0.0001$) between the selected tryptophan-modified sequences and either the standard or phenylalanine-modified versions for all samples tested. All reactions were performed in BLI binding buffer (10 mM HEPES pH 7, 150 mM NaCl, 3 mM EDTA, 0.05% Tween 20).

## References

(1) Nikoomanzar, A.; Dunn, M. R.; Chaput, J. C. Engineered polymerases with altered substrate specificity: expression and purification. *Curr. Protoc. Nucleic Acid Chem.* **2017**, *69*, 4.75.

(2) Sau, S. P.; Fahmi, N. E.; Liao, J.-Y.; Bala, S.; Chaput, J. C. A scalable synthesis of α-L-threose nucleic acid monomers. *J. Org. Chem.* **2016**, *81*, 2302-2307.

(3) Liao, J.-Y.; Bala, S.; Ngor, A. K.; Yik, E. J.; Chaput, J. C. P(V) Reagents for the Scalable Synthesis of Natural and Modified Nucleoside Triphosphates. *J. Am. Chem. Soc.* **2019**, *141*, 13286-13289.

(4) Li, Q.; Maola, V. A.; Chim, N.; Hussain, J.; Lozoya-Colinas, A.; Chaput, J. C. Synthesis and Polymerase Recognition of Threose Nucleic Acid Triphosphates Equipped with Diverse Chemical Functionalities. *J. Am. Chem. Soc.* **2021**, *143*, 17761-17768.

(5) McCloskey, C. M.; Li, Q.; Yik, E. J.; Chim, N.; Ngor, A. K.; Medina, E.; Grubisic, I.; Co Ting Keh, L.; Poplin, R.; Chaput, J. C. Evolution of Functionally Enhanced alpha-l-Threofuranosyl Nucleic Acid Aptamers. *ACS Synth. Biol.* **2021**, *10*, 3190-3199.

(6) Yik, E. J.; Medina, E.; Paegel, B. M.; Chaput, J. C. Highly Parallelized Screening of Functionally Enhanced XNA Aptamers in Uniform Hydrogel Particles. *ACS Synth. Biol.* **2023**, *12*, 2127-2134.
